# Minor compositional alterations in faecal microbiota after five weeks and five months storage at room temperature on filter papers

**DOI:** 10.1038/s41598-019-55469-0

**Published:** 2019-12-12

**Authors:** Sebastian von Huth, Louise Bruun Thingholm, Corinna Bang, Malte C. Rühlemann, Andre Franke, Uffe Holmskov

**Affiliations:** 1 Cancer and Inflammation Research, Department of Molecular Medicine, University of Southern Denmark, J. B. Winslows Vej 25.3, DK-5000 Odense C, Denmark; 2 Institute of Clinical Molecular Biology, Christian Albrechts University of Kiel, Rosalind-Franklin-Str. 12, 24105 Kiel, Germany

**Keywords:** Next-generation sequencing, Metagenomics, Microbiome

## Abstract

The gut microbiota is recognized as having major impact in health and disease. Sample storage is an important aspect to obtain reliable results. Mostly recommended is immediate freezing, however, this is not always feasible. Faecal occult blood test (FOBT) papers are an appealing solution in such situations, and most studies find these to be applicable, showing no major changes within 7 days storage at room temperature (RT). As fieldwork often requires RT storage for longer periods, evaluation of this is warranted. We performed 16S rRNA gene sequencing of 19 paired faecal samples immediately frozen or kept five weeks and five months at RT on FOBT papers. Alpha-diversity evaluation revealed no effect of FOBT storage, and evaluation of beta-diversity showed that host explained 65% of community variation, while storage method explained 5%. Evaluation of community dispersion and the Firmicutes/Bacteroidetes ratio revealed a larger effect of storage time for fresh-frozen samples. Single taxa evaluation (order-to-genus level) showed significant alterations of four (of 37) genera after five weeks and five genera after five months. When comparing the two timepoints, alterations were only detectable for fresh-frozen samples. Our findings reveal that long term storage on FOBT papers is an applicable approach for microbiota research.

## Introduction

There is an increasing attention towards the human microbiota, i.e. the collection of microbes that inhabit internal and external surfaces of the human body. Over the past decade, our understanding of the microbiota and its impact on health and disease has expanded immensely. The human gut microbiota is the most widely studied, and has been linked to a wide range of both intestinal and extra-intestinal conditions, including inflammatory bowel diseases^1,2^, colorectal cancer^3,4^, autoimmune^5^ and metabolic diseases^6^, mental illnesses^7^, obesity^8,9^ and cardiovascular diseases^10,11^. Evidently, the gut microbiota modulates both innate and adaptive immune responses within the host^12–14^, and is crucial for host physiology and defense^15^.

Uniform and suitable sample collection and storage procedures are crucial for clear and unbiased evaluation of the microbiota. Faecal samples reflect the luminal bacterial composition of the distal colon, and is, due to its non-invasive nature, an acceptable proxy for sampling the distal gut microbiota^16^. As microbial DNA might be degraded by oxidation, hydrolysis or enzymatic degradation^17^, sampling and storage methods are important factors in microbiota studies. Further, compositional changes of the microbiota can be observed due to deviating abilities of taxa to survive and replicate when exposed to oxygen. In the literature, it is claimed that storage of faecal samples at room temperature (RT) may induce alterations within 24 hours from defecation^18^. Thus, rapid freezing to −80 °C is generally considered best-practice in cases where immediate DNA extraction is not possible^19^, and is, among others, recommended by the Human Microbiome Project^20^. Some studies suggest that long-term storage at this temperature might alter the ratio between the two predominant bacterial phyla of the gut microbiota, Firmicutes and Bacteroidetes^21^.

However, rapid freezing is not always feasible, in which case long-term storage at room or ambient temperature is inevitable. This is especially true in fieldwork in remote locations, or in regions, where electrical supply is unstable or freezing capacity is limited. Different preservation kits and buffers, including faecal occult blood test (FOBT)-cards and RNA preservation reagents have been used to extend storage time at RT prior to DNA extraction and investigation of microbiota composition^22,23^. Although RNA preservation reagents have proven to be a convenient solution to stabilize the microbiota for up to two weeks at RT^18^, some studies find that this preservative performs poorly in microbiota-related studies^24,25^. Additionally, this and other preservatives are often costly, and by so, not feasibly for larger studies. Further, concerning field studies, the transportation of liquid biohazardous samples to central laboratory facilities can be cumbersome. Thus, the use of storage on FOBT cards without any liquid additives has gained ground. The majority of studies examining the use of FOBT-cards in microbiota research conclude that storage-induced differences are minor, compared to intra-individual differences^24–28^. However, these studies do investigate storage time at RT on FOBT-cards for up to 7 days, which might not be adequate time to reach freezing facilities in fieldwork.

We aimed to verify that FOBT-cards are useful as a long-term storage method in microbiota research, and further to investigate if five weeks to five months storage time on FOBT-cards at RT induce alterations in the faecal microbiota. By 16S rRNA gene sequencing, we performed pairwise comparisons of bacterial diversity and composition of faecal samples from 22 healthy individuals. One part was immediately frozen and kept at −80 °C, and one part kept on FOBT-card. Both parts were kept for five weeks (denoted timepoint 1 (TP1)) and five months (denoted timepoint 2 (TP2)), respectively, at either −80 °C or room temperature. Technical variability was examined, and sequencing data from the conventionally stored sample, which was immediately frozen, was regarded as reference.

## Results

### Study participants

Faecal samples were collected from 22 healthy normal-weighted individuals (59% males), free of any medication 2 months prior to inclusion. Median age (at time of sampling) was 30.9 years (range 23–64) and mean body mass index (BMI) was 22.3 kg/m^2^ (range 19.4–26.6). Sample-pairs from 19/22 participants successfully underwent 16S rRNA sequencing with a minimum read count of 10,000 post quality control, whereas either DNA extraction or sequencing was unsatisfactory in 3/22. An overview of cohort characteristics is provided in Table 1.

**Table 1:** Overview of study participants.

	Males		Females	
No., n (%)	13	(59%)	9	(41%)
Age, mean (range)	36.3	(23.6–64.8)	37.6	(25.4–59.6)
BMI, mean (range)	23.0	(20.3–26.6)	21.2	(19.4–24.2)

22 participants were included, from which paired samples (conventionally stored at −80 °C and sample stored five weeks (TP1) and five months (TP2) at room temperature) were prepared. 3/22 paired samples had unsatisfactory DNA extraction or 16S rRNA sequencing and were excluded from downstream analysis.

### Microbiota diversity within faecal samples

**Figure 1:**
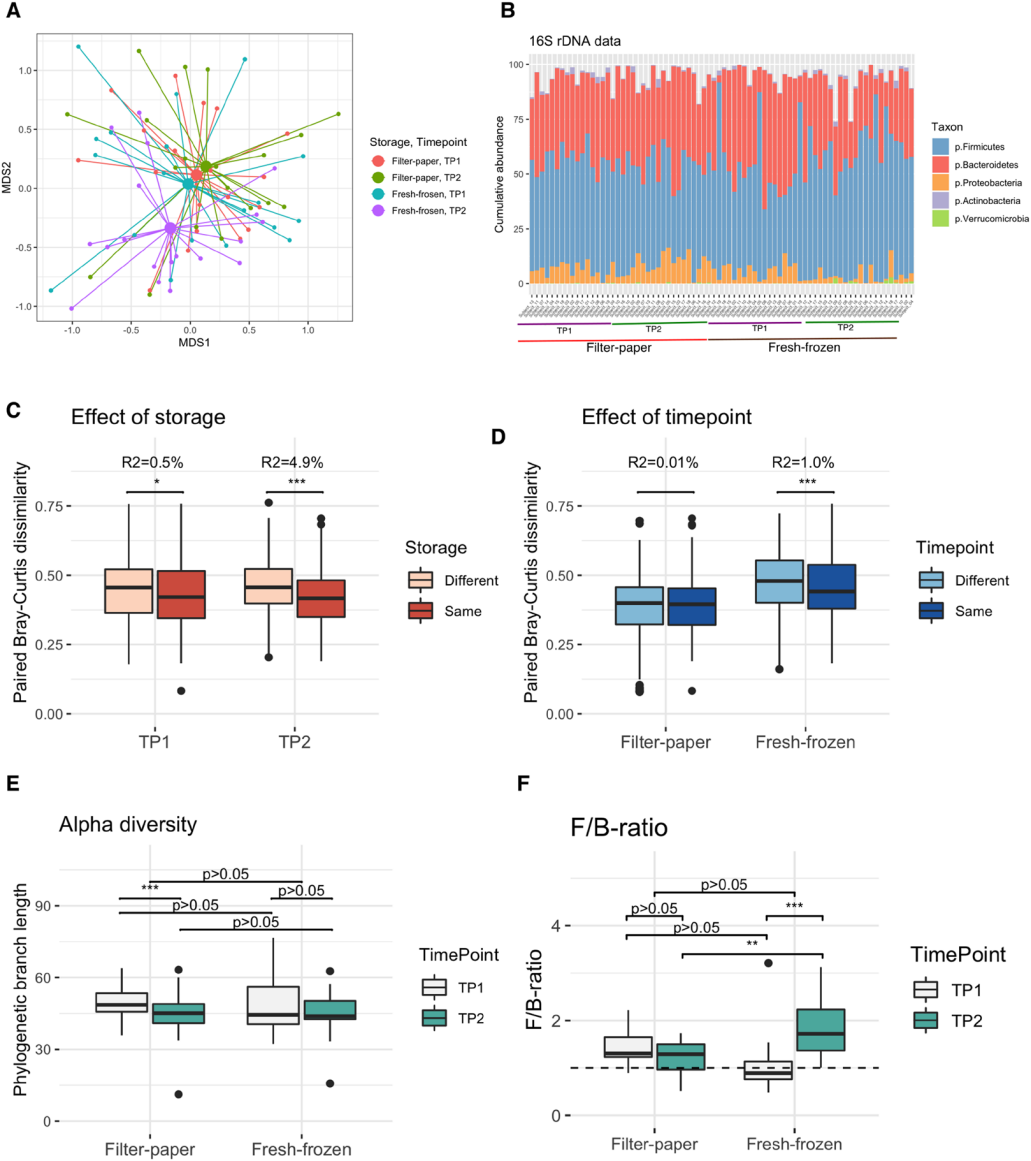
Storage method introduced a small but significant variation in microbiota diversity. (**A**) Bray-Curtis dissimilarity based MDS plot of genera community coloured by storage method and timepoint (red (TP1) and green (TP2) for filter paper; turquoise (TP1) and purple (TP2) for fresh-frozen) with individual samples and centroids connected for each group. The panel highlight the shift in location of fresh-frozen samples at TP2. (**B**) Relative abundance of core phyla across 76 samples (19 subjects) clustered by storage method (fresh-frozen and filter-paper samples). Five most abundant classes are annotated and coloured as listed. (**C**) Dissimilarity between samples stored with same (dark red) or different (light red) storage methods, calculated using Bray-Curtis dissimilarity and vegdist in R package vegan. The panel highlight a larger dissimilarity between samples stored using different methods as compared to same method. Results of a linear mixed model is given above the boxes with significance and variation explained (R^2^) (Methods). * for 0.01 =  < p < 0.05, ** for 0.001 =  < p < 0.01, *** for p < 0.001. (**D**) Dissimilarity between samples stored for the same time duration (dark blue) or different time durations (light blue), calculated as in C. The panel highlight a slightly lover dissimilarity between samples stored using filter-paper as compared to fresh-frozen storage, and a slight but significant difference between fresh-frozen samples stored for 5 weeks versus 5 months. Statistics were calculated and assigned as for C. **(E)** Phylodiversity as a measure of alpha diversity was calculated for all 76 samples and compared between storage methods (left and right) and storage duration (TP1 = grey, TP2 = green). A significant effect of storage duration could be detected (paired Wilcoxon signed rank test, p < 0.05) for filter-paper samples, while there was no difference between storage duration for fresh-frozen samples or between storage methods, joined or separated by timepoint. **(F)** Comparison of the F/B-ratio showing no significant difference between storage methods when considering both timepoints (paired Wilcoxon signed rank test, p > 0.05), however showed a difference at TP2 (p < 0.05). When comparing F/B-ratio between timepoints within storage method, a significant difference was detected for the fresh-frozen samples (p < 0.05). For panel A and B, the corresponding plot based on Jaccard similarity is found in Supplementary Fig. 1.

**Figure 2:**
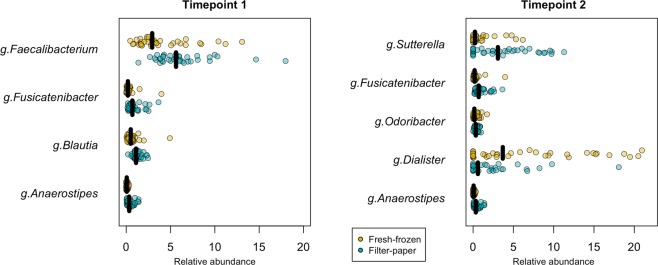
Limited alteration in relative abundance of single taxa after five weeks and five months at RT on filter-paper. Relative abundance of genera significantly altered after five weeks (TP1, left panel), and five months (TP2, right panel) storage at −80 °C (fresh-frozen, yellow) or at RT (filter-paper, blue) (Wilcoxon adjusted p < 0.05). Summary statistics are found in Supplementary Table 1 together with results for core taxa at family, class and phyla level. Differences were tested using Wilcoxon signed rank test and ANCOM.

The effects of RT storage on microbiota diversity was evaluated by pairwise comparisons of dissimilarity (Bray-Curtis and Jaccard), homogeneity of groups dispersions, sources of variability, alpha-diversity, the ratio between the two dominant phyla (Firmicutes-to-Bacteroidetes (F/B)-ratio) (Fig. 1), and single taxa relative abundance (Fig. 2 and Supplementary Table 2).

Evaluation of the microbiota composition, using Bray-Curtis and Jaccard to compare the microbiota between samples, highlighted a similar composition at TP1 and TP2 for the filter-paper samples, which all clustered together with the fresh-frozen samples at TP1. In comparison, the fresh-frozen samples at TP2 appeared shifted away from the three co-located subgroups (capscale MDS plot, Figs. 1A and Supplementary Fig. 1). The relative abundance of main phyla in Fig. 1B supported the observed stability across timepoints for filter paper, and further indicated a decreased diversity in samples stored using filter paper as compared to fresh-frozen samples. In agreement with this observation, we found the effect of storage at TP1 to be minimal (Linear mixed model, Bray-Curtis, R^2^ = 0.5%, p < 0.05), and less than the effect of storage at TP2 (R^2^ = 4.9%, p < 0.05, Fig. 1C, Supplementary Table 2). When comparing the dissimilarity of microbiota composition between samples at the same versus different timepoints (within storage type), we found no significant difference for filter-paper samples (Linear mixed model, Bray-Curtis, R^2^ = 0.014%, p > 0.05), while there was a small but significant difference for fresh-frozen samples (R^2^ = 1.0%, p < 0.05, Fig. 1D, Supplementary Table 2). The corresponding results for Jaccard similarity are found in Supplementary Table 2.

Finally, we found that dispersion for filter-paper samples was reduced compared to conventionally stored samples at TP1, while there was no significant difference at TP2 (betadisper, Bray-Curtis, p < 0.05 and p > 0.05, respectively, Supplementary Fig. 1, Supplementary Table 2). When comparing dispersion between timepoints, we found a slight but significant difference for fresh-frozen samples, but no difference for filter-paper samples (betadisper with permutest for significance, Bray-Curtis, p < 0.05 and p > 0.05, respectively). Thereby, evaluation of beta-diversity as a measure of dissimilarity in microbiota composition showed a small effect of storage-method, with a larger difference after 5 months of storage. However, we found a larger effect of 5 months of storage on the fresh-frozen samples compared to filter-paper storage. Filter-paper storage appeared to capture a slightly smaller amount of diversity as compared to fresh-frozen after five weeks of storage, however the difference was eliminated after 5 months of storage by a decrease in the diversity of the fresh-frozen samples. The corresponding analysis using Jaccard supported the results (Supplementary Table 2**)**.

Considering phylogenetic diversity, as a measure of alpha-diversity, we found no difference between the conventionally stored samples and the FOBT-samples when considering all samples at both timepoints, and when comparing storage methods at each timepoint separately (Wilcoxon p > 0.05, Fig. 1E). While there was no difference between storage methods, there was a significant difference between the two timepoints for filter-paper samples (Wilcoxon p < 0.05, Fig. 1). Phylogenetic diversity in each sample is available for visual inspection in Supplementary Fig. 4A.

A previous study suggested that the F/B-ratio was affected by storage method^29^, and we therefore evaluated if there was a significant difference in the current study. We found no significant difference between the two storage methods when considering all samples (Wilcoxon p > 0.05) or between storage methods considering samples from TP1 (p > 0.05). However, there was a significant difference between storage methods at TP2 (p < 0.05), as well as between timepoints for fresh-frozen samples (p < 0.05). This difference was not observed for filter-paper samples (p > 0.05). F/B-ratio in each sample is available for visual inspection in Supplementary Fig. 4B.

In order to defend the use of filter paper when immediate freezing is not feasible, it is essential that the individual or sampling site is the dominating source of variation, and that it dominates over the variation originating from storage type and time. To evaluate the sources of variability, we calculated Bray-Curtis dissimilarity and estimated the percentage of microbial variability that was explained by subject while correcting for storage type and timepoint. Using a linear mixed model for the data separated into the two timepoints, we found that subject explained 32% and 65% of the variation in the microbiota community at TP1 and TP2, respectively (lmer in R package lme4^30^ analysing Bray-Curtis pairwise dissimilarities, including fixed effect term for storage and mixed effect term for subject, using r.squaredGLMM in R to calculate R^2^ values). The model found variation explained by storage to be 0.5% and 4.9% at timepoint 1 and 2, respectively. Therefore, both variables accounted for variation in the microbiota community, but the far majority of variation originated from subject. The corresponding analysis with Jaccard gave very similar results, as seen in Supplementary Table 2. To further evaluate these findings, we performed hierarchical clustering analysis at the two timepoints. The analysis supported the observation that subject is the main driving factor of diversity with subject-distinctive clusters forming for 12 of 19 subjects at TP1 and 14 of 19 subjects at TP2 (hclust function in R package stats, Bray-Curtis and average agglomeration method, square root transformed genera, Supplementary Fig. 3).

### Specific taxa alterations upon storage at room temperature

Single taxa at the genera, family and class level were evaluated for association with storage method using a both paired Wilcoxon signed rank test and analysis of composition of microbiomes (ANCOM). ANCOM was included as a supportive analysis, as this method accounts for the underlying structure in microbiota data, and is superior to control for false positive inflation. Significant differential taxa were selected as those detected using both methods (Wilcoxon adjusted p < 0.05 and a true detection with cut-off 0.6 for ANCOM). We found two genera (out of 37 core genera) to significantly deviate between storage methods at TP1 and one genus deviating at TP2 (Fig. 2, Supplementary Table 1). At TP2, we found two family and two class-level taxa to deviate in relative abundance between storage methods, while no single taxa differed at TP1 (Supplementary Table 2). Negativicutes and Betaproteobacteria were the two classes found to deviate at TP2, a finding further supported by Supplementary Fig. 1B.

For all samples, the most dominant classes were Clostridia and Bacteroidia, followed by Negativicutes and Betaproteobacteria. At phyla level, Proteobacteria were found significantly decreased in fresh-frozen samples at TP2, while no single phyla were differentially abundant at TP1.

To evaluate if storage time affected filter-paper samples more or less than samples stored conventionally, we compared the abundance of core taxa between timepoints for each storage method. Again, taxa were detected as differentially abundant if both analyses supported the association (Wilcoxon adjusted p < 0.05 and a true detection with cut-off 0.6 for ANCOM, respectively). No genera or family-level taxa were found to be significantly altered by storage time in the neither fresh-frozen samples nor in the filter-paper stored samples (Wilcoxon detected two genera, *Anaerostipes* and *Blautia*, to associate with storage time for fresh-frozen samples, adjusted p < 0.05, Supplementary Table 1). A similar pattern was observed at the class level, where two taxa were significantly altered by storage time in the fresh-frozen samples while no single taxa appeared significantly altered in samples stored using filter-paper (Wilcoxon detected association of five taxa with storage time for fresh-frozen samples, adjusted p < 0.05, however the association was not supported by ANCOM). At phyla level, Bacteroidetes was significantly altered by storage time in fresh-frozen samples while no single phyla were altered in samples stored on filter paper.

## Discussion

Sample collection and storage are important aspects in microbiota research for accurate analysis and obtaining reliable and reproducible results. Although current recommendations recommend storage at −80 °C, this is not possible in fieldwork or large-scale studies where at-home sampling is preferred. In the current study, we investigated whether the microbiota from faecal samples stored on FOBT-cards for five weeks to five months at RT would have any alterations compared to rapid frozen and conventionally stored samples. By high-quality sequencing of the 16S rRNA gene, we compared dispersion, diversity and F/B ratio of the two storage methods, and further analysed compositional changes. We did not find significant differences in alpha diversity or F/B-ratio and found limited single- taxa differences of core taxa at class, family or genera level of the intestinal microbiota, when comparing paired samples from 19 individuals.

Importantly, we demonstrate only minor compositional differences in gut microbiota due to storage, and further validate that the microbiota of filter papers reflects that of conventionally stored samples and that any changes induced by the storage method are minor. Out of 37 identified core genera, we found that the relative abundance of only two genera, all from the Clostridiales order, increases upon five weeks storage at room temperature (Supplementary Table 1**)**. The genera in question are abundant in the healthy gut microbiota^31^, and are important in dietary fibre digestion and production of butyric acid^32^. Although we cannot exclude contamination of the utilized FOBT papers, we are rather confident that these alterations arise from the storage procedure, and that the genera are not a part of the FOBT papers.

One major concern of storing faecal samples at room temperature is the fact that some bacterial taxa common to the gut, predominantly members of the Proteobacteria phylum, are able to grow in oxygen at room temperature^33^. Such facultative anaerobic bacteria may confound the analysis, as overgrowth might not reflect physiological alterations that were present prior to sampling, but instead resemble continued growth following sampling. A recent meta-analysis of storage conditions demonstrated a consistent trend in bacteria growing at room temperature (termed “blooming bacteria”), of which most belonged to the Gammaproteobacteria class^33^. In the present study, we do not find any alterations in the Gammaproteobacteria class at TP1 or TP2. However, at TP2 we find increased levels of *Sutterella* (using the Wilcoxon signed rank test), a genus from the Betaproteobacteria class, and Betaproteobacteria itself increased in filter-paper samples (p < 0.05, Supplementary Table 1**)**. This finding indicates no specific selection of facultative anaerobic bacteria as only one of the four genera increased at TP2 belong to the Proteobacteria phylum. This implies that facultative anaerobic bacteria are not able to continued growth following storage at RT on FOBT paper, as would be one of the main concerns regarding this storage method.

Our findings are in line with previous publications and supports the use of FOBT-cards for sample storage in microbiota research projects, where immediate freezing is not possible. Further, our results might suggest that samples stored at filter paper may preserve some aspects of the microbiota structure better than fresh frozen samples. Although we identify some alterations induced by storage, these are all minor. FOBT cards are suitable for storage for up to five months, without limitations to sequence data quality.

## Methods

### Study subjects, sample collection and storage

Faecal samples were collected from 22 healthy individuals in January 2018. Although we aimed to limit the number of covariates known to profoundly affect the gut microbiota, we assessed intra-individual differences due to storage, which limits the importance of a homogenous cohort. None of the participants had received any antibiotics or other medication two months prior to inclusion, and none reported any gastrointestinal complaints. Female subjects were not pregnant. Faecal samples were collected in sterile faeces tubes (Sarstedt, Nümbrecht, Germany), and handled within 15 minutes from defecation. The faecal matter was manually homogenized, and approximately 0.5 mL was subsequently applied to two faecal occult blood test (FOBT) filter card (Hemoccult®, Beckman Coulter Inc., Brea, CA, USA) with a clean wooden spatula, and left to dry under laminar airflow for 2–6 hours, protected from light. The remaining faecal sample was immediately frozen and kept at −80 °C. The filter paper samples were kept in air-tight zip-lock bags with desiccant (Whatman® desiccant packs, Sigma-Aldrich, Schnellendorf, Germany), and stored at room temperature protected from light for five weeks and five months, respectively, after which the samples were moved to −80 °C, prior DNA extraction.

### DNA extraction and quality control

Filter papers from FOBT-card samples were handled with ethanol-cleansed instruments (EMSURE®, Merck, Darmstadt, Germany), and DNA was extracted using the QIAamp DNA Stool Mini Kit (QIAGEN, Hilden, Germany) on a QIAcube platform (QIAGEN), according to manufacturer’s instructions with minor modifications. Briefly, the filter paper underwent bead-beating in PowerBead Tubes with Garnet beads (QIAGEN) on a SpeedMill PLUS instrument (Analytik Jena AG, Jena, Germany), prior to QIAcube extraction. DNA from conventionally stored samples were extracted using the QIAamp DNA Stool Mini Kit (QIAGEN) on a QIAcube platform (QIAGEN), according to manufacturer’s instructions. Extracted DNA was stored at −80 °C prior to PCR amplification. Blank extraction controls were included during extraction of samples. DNA quality from all samples was evaluated by standard gel analysis, to identify possible fragmentation of genomic DNA.

### PCR and 16S rRNA sequencing

PCR amplification and 16S rRNA sequencing was performed as described by Wang *et al*.^34^. In brief, the V1-V2 regions of the 16S rRNA gene were amplified as described by Kozich *et al*.^35^, using the 27F-338R primer pairs, and further dual-barcoded, as described by Caporaso *et al*.^36^. Sequencing was performed on the Illumina MiSeq platform (San Diego, CA, USA), according to manufacturer’s instructions.

### 16S rRNA data processing

Sequencing was performed on the Illumina MiSeq platform, using the MiSeq Reagent Kit v3 according to manufacturer’s instructions. MiSeq FastQ files were trimmed using sickle^37^ in PE (paired-end) mode with a sliding window of 0.1 readlength. Trimming was performed when average quality within the window was below 20, and reads were all >100 bp after trimming. Reads were stitched using VSEARCH^38^ with a length between 280 and 350 bp. Further, VSEARCH filtered reads with more than 1 expected error. Further quality filtering was performed using the FastX-Toolkit::fastq_quality_filter^39^ to exclude sequences with >5% nucleotides with a quality score below 30. Files were subsequently converted to FASTA format, and chimeras were removed in VSEARCH, using the gold.fa database. The remaining reads were classified using the UTAX algorithm, where reads classified as chloroplasts or not classified at domain level were removed.

OTU tables were generated in UPARSE^40^, implemented in VSEARCH. After removal of replicates and singletons, reads were clustered based on 97% similarity. Chimeras were once again filtered using VSEARCH in de-novo mode. To generate OTU abundance tables, all reads per sample were mapped to OTU tables using VSEARCH. Using the SINTAX classifier^41^ at lowest possible level with minimum 80% bootstrap confidence, one representative sequence for each OTU was annotated. OTUs with identical annotations were grouped into taxonomic bins.

Samples with fewer than 10,000 reads were removed (n = 9) as were subjects with < 4 samples post processing leaving 19 samples (76 samples) for further analysis.

### Bioinformatics and statistical analysis

The R programming environment v3.5.1^42^ was used for statistical analysis of the microbiota data, and adjusted p-values (P.adj) were obtained using the p.adjust function in R package stats v3.5.1 with Benjamini-Hochberg (method = “BH”).

Profiles for beta-diversity and single taxa analysis was transformed to adjust for deviating sequencing depth by dividing the counts by sample sum and multiply by 100 to obtain relative abundances between zero and 100.

The alpha diversity measure considered was phylodiversity, as a measure of total unique phylogenetic branch length (calculated using mothur’s phylo.diversity function with the phylogenetic tree built using FastTree with–nt and–gtr and the 16S OTU table as input).

The profiles for single taxa analysis were filtered to keep most abundant taxa as follows; minimum mean abundance across all samples of 0.05, and a minimum abundance of 0.05 in at least one sample. Further, taxa with ≥40% none-zeroes across samples were removed, which left 11 classes, 19 families and 37 genera. The relative abundance of single taxa was evaluated at phyla, class, family and genus level.

Analyses of F/B-ratio and alpha diversity were performed using the non-parametric paired Wilcoxon signed rank test in R package stats with conf.int = TRUE to obtain information of the location shift. Analyses of single taxa were performed using the described Wilcoxon signed rank test. However, as this test might result in high false positives, we supplied the analysis of differential abundances with the analysis of composition of microbiomes (ANCOM)^43^. This method accounts for the underlying structure in microbiome data and may therefore be superior in controlling for false positive inflation. ANCOM analysis was performed using abundance profiles with count data, not rarefied, and only including taxa selected for Wilcoxon analysis. ANCOM settings were adjusted = F, repeated = F, multcorr = 2, sig = 0.05 and prev.cut = 0.90. Due to the low sample size and often non-normality of the data we deemed this test appropriate.

Analysis of dispersion was performed using the betadisper function in R package vegan^44^ (type = “centroid”). Significance of the difference in dispersion between groups was estimated using the anova function in R package stats.

Evaluation of the association between storage method, storage time or host and microbial community structure was performed using linear mixed-effects models (LMM) in the lmer function in R package lme4^30^ with default settings. LMM was used to allow specification of the data structure with multiple samples from same subject. Association with storage time: Bray-Curtis pairwise dissimilarities were calculated between all samples stored with same storage method. For each distance measure, ids for the compared subjects and timepoints (same or different) were generated. Then a model was constructed including timepoint variables as fixed effect and intercepts for subject-pair variables as random effects. For association with storage method, pairwise dissimilarities were calculated between all samples stored for the same time duration. For each distance measure, ids for the compared subjects and storage method (same or different) were generated. Then data was separated according to timepoint and a model constructed including storage method variables as fixed effect and intercepts for subject-pair variables as random effects. The described analysis for storage time and method was also performed using Jaccard similarity measure. The r.squaredGLMM function in R package MuMIn^45^ was used to calculate conditional (R2c) and marginal (R2m) coefficient of determination. Variance explained by subject (R^2^) was calculated as R2c (variance explained by the entire model) minus R2m (variance explained by the fixed effects). Finally, hierarchical clustering was evaluated using the hclust function in R package stats, with Bray-Curtis, average agglomeration method, and square root transformed genera profiles.

### Additional R-packages used

reshape2 v1.4.3^46^, grid v3.5.1, gridExtra v2.3^47^, gridBase v0.4.7^48^, gridGraphics v0.3.0^49^, plyr v1.8.4^50^, ggplot2 v3.1.1^51^, ggpubr v0.2^52^, ggsignif v0.4.0^53^, extrafont v0.17^54^, scales v1.0.0^55^, vegan v2.5.4^44^, lmerTest v3.0.1^56^, lme4 v1.1.19^30^, metafor v2.0.0^57^, genefilter v1.64.0^58^, MuMIn v1.42.1^45^, dendextend v1.12^59^.

### Ethical statement

Individuals were included voluntarily, and informed consent was obtained. The study was approved by the Regional Ethics Committee of Region of Southern Denmark (reference no. S-20170165) and conducted according to the principles of the Declaration of Helsinki.

## Supplementary information

Supplementary information for the manuscript

## Data Availability

The complete dataset including raw reads and anonymised metadata is available on the NCBI SRA repository (Accession no. PRJNA550519).
