# Supplementary material for: Minor compositional alterations in faecal microbiota after five weeks and five months storage at room temperature on filter papers
Source: Sci Rep. 2019 Dec 12;9:19008. doi: 10.1038/s41598-019-55469-0 (PMC6908594; doi:10.1038/s41598-019-55469-0)

SUPPLEMENTARY INFORMATION FOR THE MANUSCRIPT

Minor compositional alterations in faecal microbiota after five weeks and five months storage at room temperature on filter papers (SREP-19-24101).

Authors

Sebastian von Huth[1,3], Louise Bruun Thingholm[2,3], Corinna Bang[2], Malte C. Rühlemann[2], Andre Franke[2], Uffe Holmskov[1*]

Author affiliations

[1] Cancer and Inflammation Research, Department of Molecular Medicine, University of Southern Denmark, J. B. Winslows Vej 25.3, DK-5000 Odense C, Denmark.

[2] Institute of Clinical Molecular Biology, Christian Albrechts University of Kiel, Rosalind-Franklin-Str. 12, 24105 Kiel, Germany.

[3] These authors contributed equally to this work and share first authorship.

* Corresponding author.

Correspondence to uholmskov@health.sdu.dk

File list:

- **Supplementary Table 1:** Summary statistics for analysis of alpha-diversity, Firmicutes/Bacteroidetes-ratio, pairwise dissimilarity, and dispersion.
- **Supplementary Table 2:** Altered taxa upon five weeks and five months storage at RT.
- **Supplementary Figure 1:** Jaccard similarity based MDS and relative abundance of core class-level taxa.
- **Supplementary Figure 2:** Dispersion between storage methods and storage duration.
- **Supplementary Figure 3:** Hierarchical cluster analysis of paired fresh-frozen and filter-paper stored samples from 19 subjects after 5 weeks and 5 months of storage.
- **Supplementary Figure 4:** Alpha diversity and F/B-ratio for each sample across subjects.

Supplementary Table 1: Altered taxa upon five weeks and five months storage at RT. Single taxa at the genera, family, class and phyla levels were evaluated for association with storage method (**A**) and storage time (**B**) using a both paired Wilcoxon signed rank test and analysis of composition of microbiomes (ANCOM). All taxa with Wilcoxon adjusted p<0.1 are listed along with results from ANCOM. **A)** Four genera, marked with red font, are significantly altered by RT storage for five weeks (TP1) on FOBT-cards, and five genera are significantly altered after five months (TP2) (Wilcoxon adjusted p<0.05). After five months, two family and two class-level taxa, respectively, deviated in relative abundance between storage methods, while no single taxa differed after five weeks. As indicated in **Supplementary Figure 1B**, Negativicutes and Betaproteobacteria were found to deviate at TP2. Relative abundances of associated genera are seen in **Figure 2**. **B)** Single taxa at the genera, family and class levels were evaluated for association with storage time when stored as fresh-frozen samples (left) or using filter-paper (right). While two genera and five class-level taxa deviated significantly in relative abundance between TP1 and TP2 in fresh-frozen samples, no single taxa deviated significantly in abundance between TP1 and TP2 when stored using filter-paper (Wilcoxon adjusted p<0.05).

A) Association with storage method

Level	Timepoint 1							Timepoint 2						
		Wilcoxon signed rank test				ANCOM			Wilcoxon signed rank test				ANCOM	
	Taxa	Statistic	Location shift	p-value	Adjusted p-value	Statistic	Detected shift (cutoff 0.6)	Taxa	Statistic	Location shift	p-value	Adjusted p-value	Statistic	Detected shift (cutoff 0.6)
Genus	g.Anaerostipes	188	0,277	1,14E-05	4,22E-04	32	TRUE	g.Anaerostipes	175	0,352	5,23E-04	7,96E-03	21	FALSE
	g.Blautia	186	0,783	2,67E-05	4,94E-04	23	TRUE	g.Dialister	16	-6,63	6,45E-04	7,96E-03	13	FALSE
	g.Fusicatenibacter	171	0,409	1,17E-03	1,44E-02	19	FALSE	g.Odoribacter	177	0,242	3,36E-04	7,96E-03	16	FALSE
	g.Faecalibacterium	169	2,4	1,69E-03	1,56E-02	2	FALSE	g.Fusicatenibacter	171	0,819	1,17E-03	1,08E-02	25	TRUE
	g.Clostridium_XVIII	160	0,269	7,14E-03	5,28E-02	9	FALSE	g.Sutterella	154	4,76	3,06E-03	2,26E-02	19	FALSE
	g.Collinsella	155	0,493	1,41E-02	7,45E-02	14	FALSE	g.Butyricimonas	158	0,206	9,45E-03	5,83E-02	1	FALSE
	g.Lachnospiracea_ince	155	0,518	1,41E-02	7,45E-02	2	FALSE	g.Clostridium_XlVa	35	-0,428	1,41E-02	7,45E-02	12	FALSE
Family								f.Sutterellaceae	183	4,96	7,25E-05	1,38E-03	17	TRUE
								f.Veillonellaceae	13	-6,8	3,36E-04	3,19E-03	12	TRUE
Class								c.Negativicutes	1	-6,83	7,63E-06	8,39E-05	7	TRUE
								c.Betaproteobacteria	184	5,79	5,34E-05	2,94E-04	9	TRUE
Phyla								p.Bacteroidetes	145	7,52	4,46E-02	7,43E-02	3	TRUE
								p.Firmicutes	31	-11,6	8,23E-03	2,06E-02	2	FALSE
								p.Proteobacteria	172	5,95	9,65E-04	4,83E-03	4	TRUE
								p.Verrucomicrobia	27	-0,513	6,49E-02	8,11E-02	1	FALSE

B) Association with storage time

Level	Fresh-frozen samples							Filter-paper samples						
		Wilcoxon signed rank test				ANCOM			Wilcoxon signed rank test				ANCOM	
	Taxa	Statistic	Location shift	p-value	Adjusted p-value	Statistic	Detected shift (cutoff 0.6)	Taxa	Statistic	Location shift	p-value	Adjusted p-value	Statistic	Detected shift (cutoff 0.6)
Genus	g.Anaerostipes	12	0,0931	2,67E-04	6,22E-03	21	FALSE							
	g.Blautia	13	0,496	3,36E-04	6,22E-03	18	FALSE							
	g.Akkermansia	16	0,555	7,74E-03	7,16E-02	7	FALSE							
	g.Odoribacter	160	-0,278	7,14E-03	7,16E-02	17	FALSE							
Family	f.Coriobacteriaceae	29	0,541	6,18E-03	7,35E-02	4	FALSE							
	f.Verrucomicrobiacea	16	0,555	7,74E-03	7,35E-02	4	FALSE							
Class	c.Actinobacteria	28	0,623	5,33E-03	2,84E-02	4	FALSE	c.Negativicutes	161	-2,36	6,18E-03	6,80E-02	0	FALSE
	c.Betaproteobacteria	166	-2,74	2,84E-03	2,84E-02	9	TRUE							
	c.Verrucomicrobiae	16	0,555	7,74E-03	2,84E-02	3	FALSE							
	c.Alphaproteobacteria	81	-0,0551	1,44E-02	3,96E-02	8	TRUE							
	c.Bacteroidia	153	-11	1,81E-02	3,98E-02	4	FALSE							
Phyla	p.Actinobacteria	28	0,623	5,33E-03	1,94E-02	2	FALSE							
	p.Bacteroidetes	156	-12,7	1,24E-02	2,07E-02	3	TRUE							
	p.Firmicutes	48	10,9	6,02E-02	7,52E-02	2	FALSE							
	p.Verrucomicrobia	16	0,555	7,74E-03	1,94E-02	2	FALSE							
	p.Proteobacteria	125	-2,17	2,41E-01	2,41E-01	3	TRUE							

Location shift: positive means increase in filter-paper

Supplementary Table 2: Summary statistics for analysis of alpha-diversity, Firmicutes/Bacteroidetes-ratio, pairwise dissimilarity, and dispersion. A) Phylodiversity, as a measure of alpha diversity, was evaluated for association with storage time and method using a paired Wilcoxon signed rank test. **B)** Firmicutes/Bacteroidetes (F/B) ratio was evaluated for association with storage time and method using a paired Wilcoxon signed rank test. **C)** Differences in pairwise Bray-Curtis dissimilarities between samples stored (i) with same or different storage method and (ii) for the same or different time duration, was compared using linear mixed-effects models as described in Methods. **D)** Groups dispersions (variances) were compared with groups defined by timepoint or storage method. Significance of the difference in dispersion between groups was estimated using ANOVA (Methods). SE: standard error. Sum Sq: sum of squares.

**A) Alpha diversity**	**(pseudo)medican**	**p-value**
Comparing storage methods, across all samples	-0,789	4,64E-01
Comparing storage methods, at TP1	-0,364	8,91E-01
Comparing storage methods, at TP2	-0,030	2,41E-01
Comparing timepoints, across filter-paper samples	2,694	9,65E-04
Comparing timepoints, across fresh-frozen samples	4,239	3,12E-01

**B) F/B-ratio**	**(pseudo)medican**	**p-value**
Between timepoints, across filter-paper samples	0,183	7,99E-02
Between timepoints, across fresh-frozen samples	-0,843	9,65E-04
Between storage methods, at TP1	0,313	1,82E-01
Between storage methods, at TP2	-0,593	1,41E-03
Between storage methods, across both TP1 and TP2	-0,211	1,80E-01

**C) Linear mixed-effects models (LMM)**	**Bray-Curtis**					**Jaccard**				
	**Estimate**	**SE**	**t-value**	**p-value**	**R2**	**Estimate**	**SE**	**t-value**	**p-value**	**R2**
Microbiota association with storage time, across filter-paper samples	-0,0026	0,0049	-0,544	5,86E-01	0,00014	-0,0029	0,0050	-0,584	5,59E-01	0,00014
Microbiota association with storage time, across fresh-frozen samples	-0,0238	0,0056	-4,217	2,93E-05	0,01012	-0,0238	0,0053	-4,513	8,00E-06	0,0107
Microbiota association with storage method, at TP1	-0,0164	0,0072	-2,276	2,33E-02	0,00501	-0,0184	0,0070	-2,650	8,31E-03	0,0066
Microbiota association with storage method, at TP2	-0,0462	0,0044	-10,57	<2E-16	0,04949	-0,0457	0,0043	-10,64	<2E-16	0,0498

**D) Dispersion**	**Bray-Curtis**			**Jaccard**		
	**Sum Sq**	**F-statistic**	**p-value**	**Sum Sq**	**F-statistic**	**p-value**
Between storage methods, at TP1	0,06465	15,235	1,00E-03	0,0558	14,431	0,001
Between storage methods, at TP2	0,00062	0,1367	7,02E-01	0,0009	0,1996	0,623
Between timepoints, across fresh-frozen samples	0,02133	4,5593	3,90E-02	0,0159	3,9892	0,06
Between timepoints, across filter-paper samples	0,00694	1,6933	1,77E-01	0,0065	1,5648	0,204

Supplementary Figure 1: Jaccard similarity based MDS and relative abundance of core class-level taxa. **A)** Jaccard similarity based MDS plot of genera community coloured by storage method and timepoint (red (TP1) and green (TP2) for filter paper; turquoise (TP1) and purple (TP2) for fresh-frozen) with individual samples and centroids connected for each group. The panel highlight the shift in location of fresh-frozen samples at TP2, as was also observed using Bray-Curtis dissimilarity. **B)** Relative abundance of core class-level taxa across 76 samples (19 subjects) clustered by storage method (fresh-frozen and filter-paper samples). Ten most abundant taxa are annotated and coloured as listed.

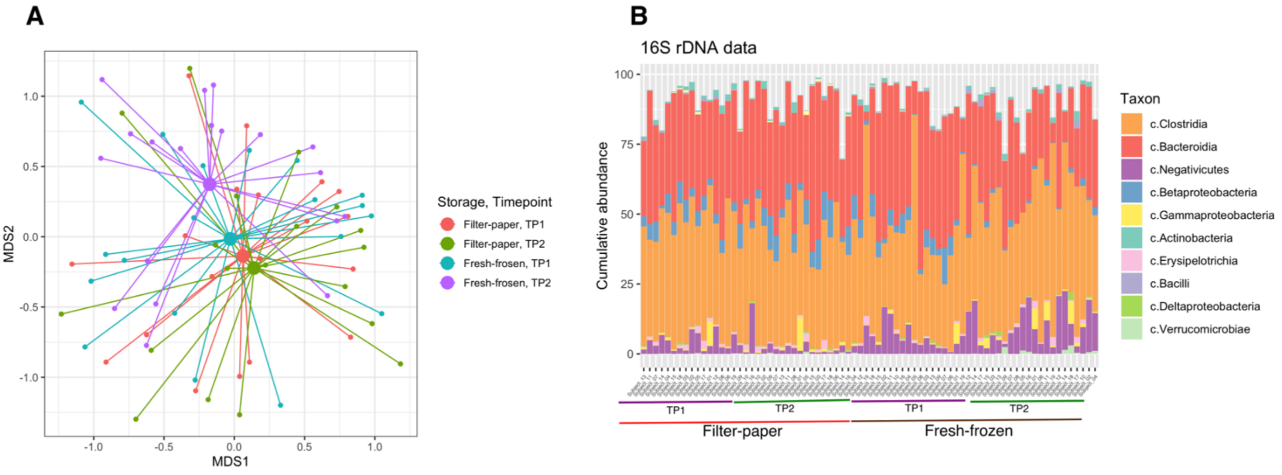

Supplementary Figure 2: Dispersion between storage methods and storage duration. Groups dispersions (variances) were evaluated using betadisper function in R package vegan (type ="centroid") with groups defined by timepoint **(A)** or storage method **(B)**. Significance of the difference in dispersion between groups was estimated using the anova function in R package stats. The analysis showed a reduced dispersion for filter-paper samples as compared to conventionally stored samples at TP1, while there was no significant difference at TP2 ($p<0.05$ and $p>0.05$, respectively). When comparing dispersion between timepoints **(B)**, we found a slight but significant difference for fresh-frozen samples, but no difference for filter-paper samples ($p<0.05$ and $p>0.05$, respectively).

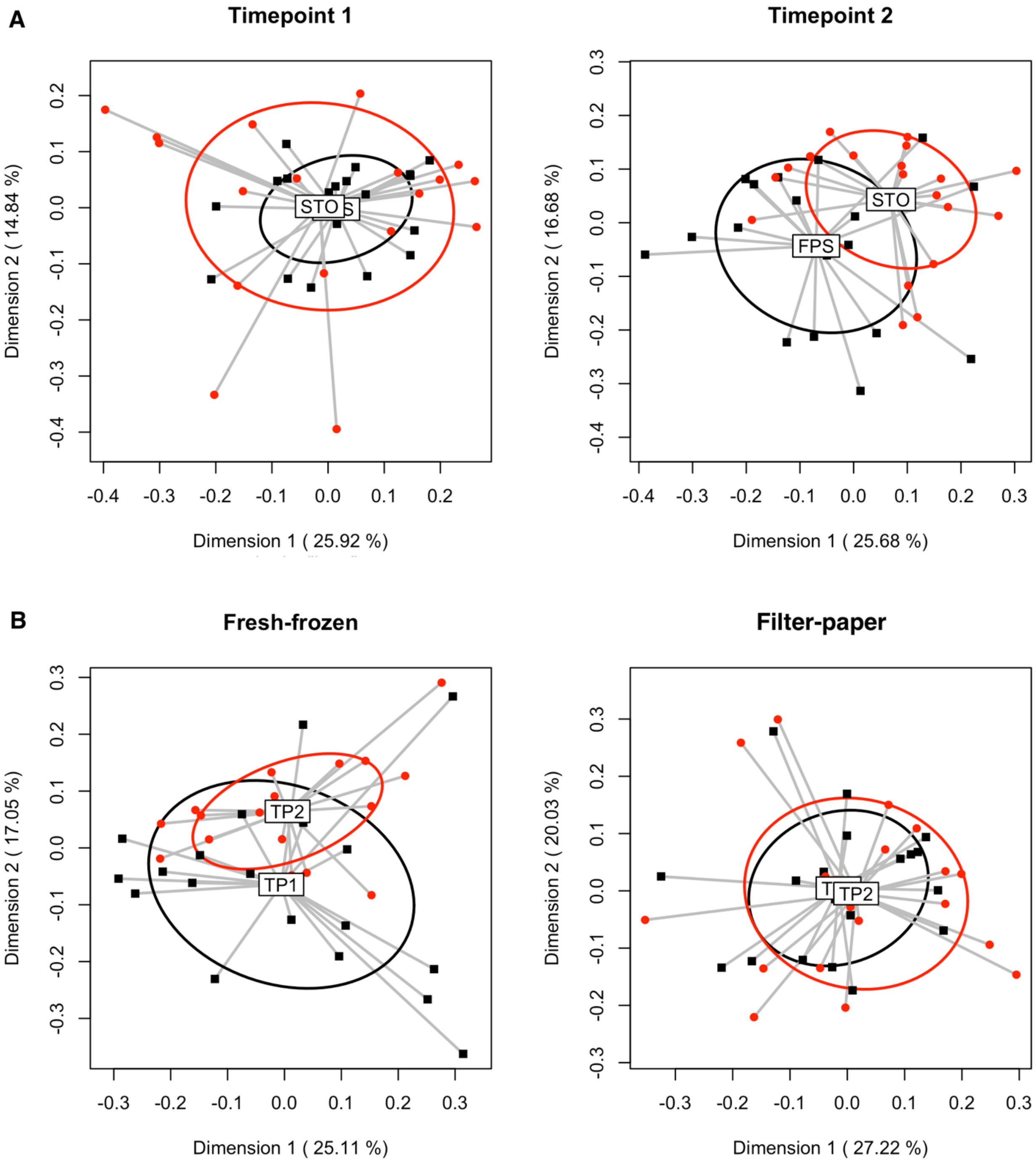

Supplementary Figure 3: Hierarchical cluster analysis of paired fresh-frozen and filter-paper stored samples from 19 subjects after 5 weeks and 5 months of storage. The analysis was performed at each timepoint, and showed subject-distinctive clusters for 12 of 19 subjects at TP1 (**A**) and 14 of 19 subjects at TP2 (**B**). The analysis was performed using the hclust function in R package stats, Bray-Curtis and average agglomeration method, square root transformed genera. The hclust object was transformed to a dendrogram using the function as.dendrogram and plotted using plot both from the R package stats. Coloured bars for subject and storage method were then added using the function colored_bars in R package dendextend.

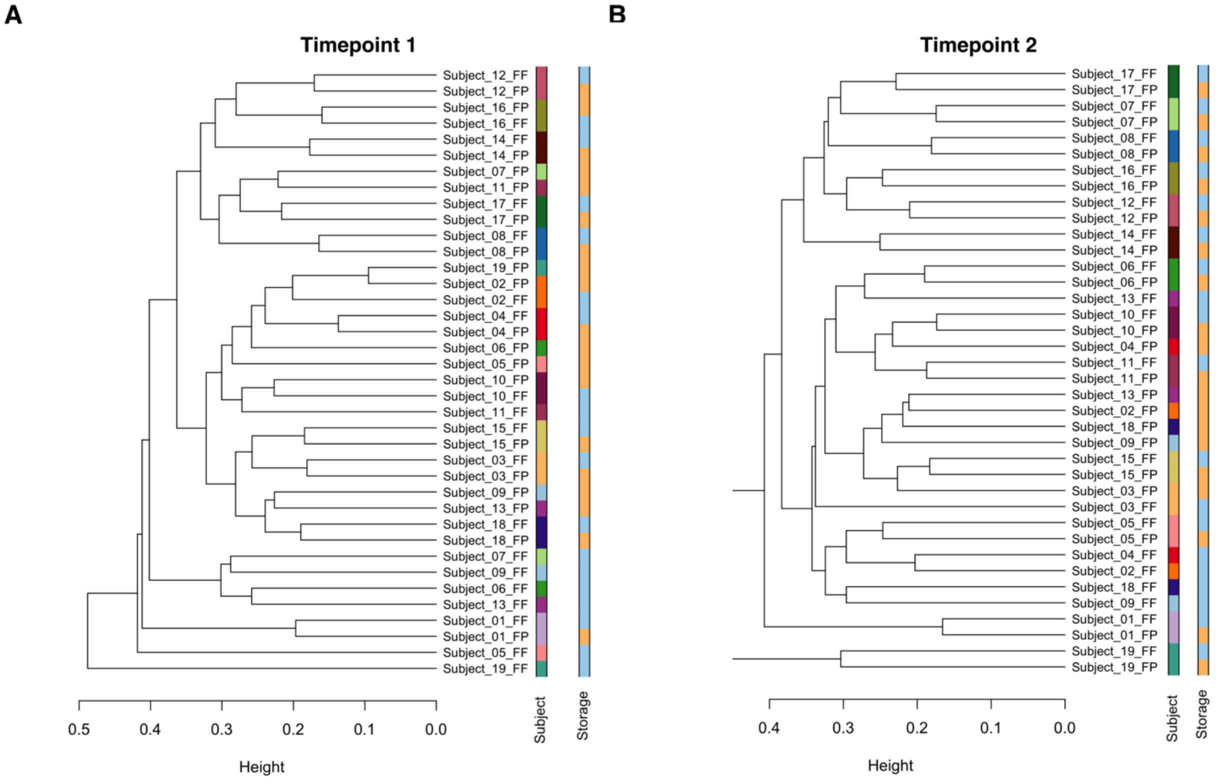

Supplementary Figure 4: Alpha diversity and F/B-ratio for each sample across subjects. A) Phylodiversity as a measure of alpha diversity across 19 subjects with one box for each sample (storage type: fresh-frozen=brown, filter-paper=grey; timepoint: TP1=light colour, TP2=dark colour) and horizontal lines indicating storage mean (fresh-frozen=brown, filter-paper=grey). **B)** Similar plot to A, however depicting the Firmicutes/Bacteroidetes (F/B) ratio across the 19 samples (colours as in panel A).

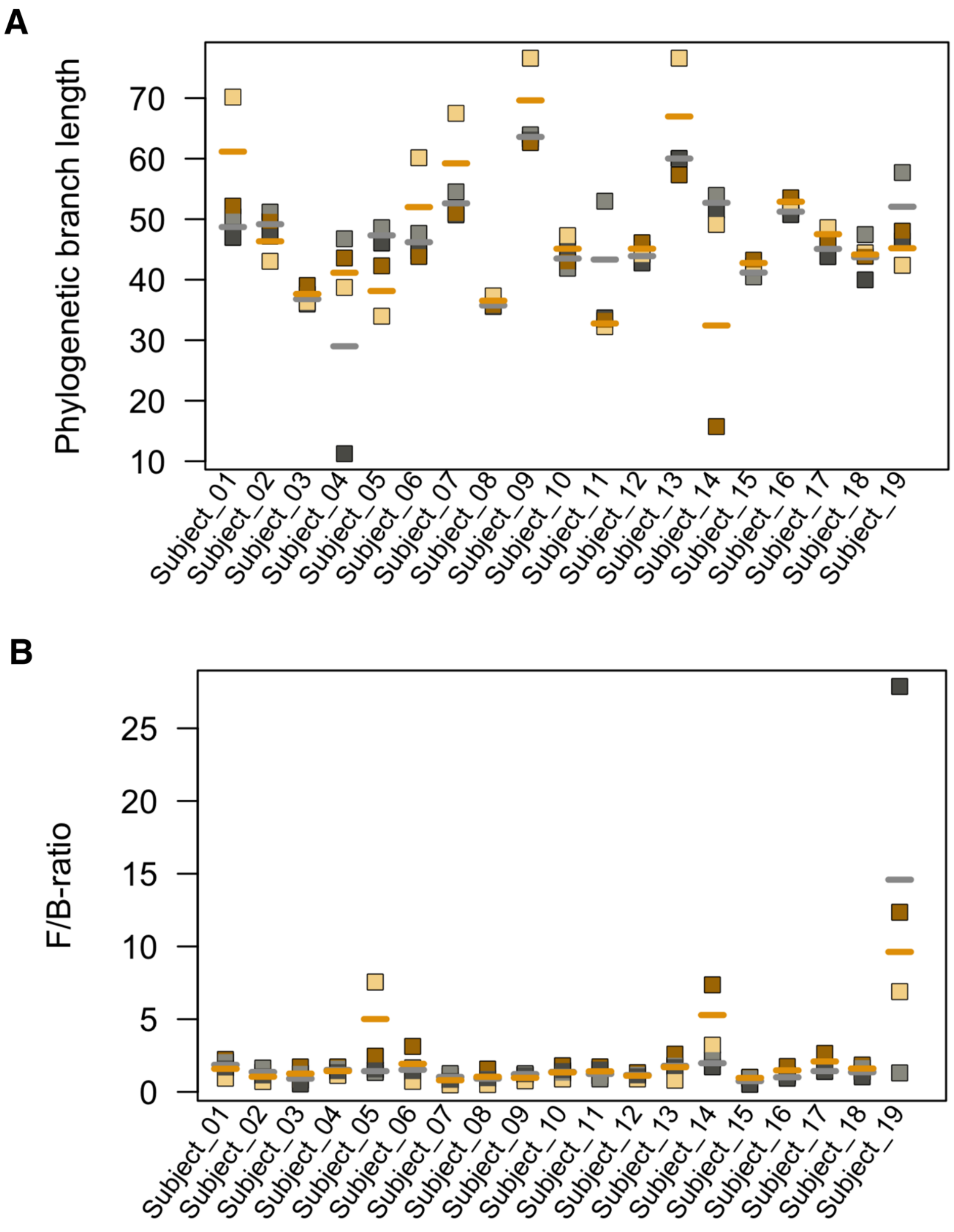

Supplement: Supplementary file 1 — Supplementary information for the manuscript [file 41598_2019_55469_MOESM1_ESM.pdf]
